# Cell wall integrity signaling regulates cell wall-related gene expression in *Chlamydomonas reinhardtii*

**DOI:** 10.1038/s41598-019-48523-4

**Published:** 2019-08-21

**Authors:** Evan Cronmiller, Deepak Toor, Nai Chun Shao, Thamali Kariyawasam, Ming Hsiu Wang, Jae-Hyeok Lee

**Affiliations:** Department of Botany, University of British Columbia, 6270 University Blvd., Vancouver, Canada

**Keywords:** Molecular engineering in plants, Biodiesel, Stress signalling, Cell wall

## Abstract

An intact cell wall is critical for cellular interactions with the environment and protecting the cell from environmental challenges. Signaling mechanisms are necessary to monitor cell wall integrity and to regulate cell wall production and remodeling during growth and division cycles. The green alga, *Chlamydomonas*, has a proteinaceous cell wall of defined structure that is readily removed by gametolysin (g-lysin), a metalloprotease released during sexual mating. Naked cells treated with g-lysin induce the mRNA accumulation of >100 cell wall-related genes within an hour, offering a system to study signaling and regulatory mechanisms for *de novo* cell wall assembly. Combining quantitative RT-PCR and luciferase reporter assays to probe transcript accumulation and promoter activity, we revealed that up to 500-fold upregulation of cell wall-related genes was driven at least partly by transcriptional activation upon g-lysin treatment. To investigate how naked cells trigger this rapid transcriptional activation, we tested whether osmotic stress and cell wall integrity are involved in this process. Under a constant hypotonic condition, comparable levels of cell wall-gene activation were observed by g-lysin treatment. In contrast, cells in an iso- or hypertonic condition showed up to 80% reduction in the g-lysin-induced gene activation, suggesting that osmotic stress is required for full-scale responses to g-lysin treatment. To test whether mechanical perturbation of cell walls is involved, we isolated and examined a new set of cell wall mutants with defective or little cell walls. All cell wall mutants examined showed a constitutive upregulation of cell wall-related genes at a level that is only achieved by treatment with g-lysin in wild-type cells. Our study suggests a cell wall integrity monitoring mechanism that senses both osmotic stress and mechanical defects of cell walls and regulates cell wall-gene expression in *Chlamydomonas*, which may relate to cell wall integrity signaling mechanisms in other organisms.

## Introduction

The cell wall is an extracellular compartment that surrounds cells and provides protection and buffering from environmental fluctuation. The morphology and composition of cell walls varies across eukaryotes, representing multiple strategies of protection^1–3^. Despite physicochemical differences, the mechanisms that monitor cell wall integrity best studied in yeast and plants are similar in terms of the mode of sensation and molecular components involved^4,5^. One common theme is the interplay between cell wall-anchored or -interacting surface receptors and osmosensing channels, which regulate the *de novo* assembly or reinforcement of existing cell walls^6^. It is, therefore, interesting to examine whether a similar or different cell wall integrity monitoring system is present outside the fungal and plant lineages.

The single-celled alga *Chlamydomonas reinhardtii* constantly builds and modifies its cell walls throughout its life cycle^7^. Occasionally, when two nitrogen-starved sexual gametes encounter each other, they initiate a ‘mating reaction’ and remove their cell walls in preparation for cell fusion and subsequent zygotic wall assembly^8^. Consequently, the cells become naked and exposed to their environment and immediately rebuild their cell walls. A failure to do so may lyse the cells in the hypotonic freshwater environments where *C*. *reinhardtii* live. Given this importance of cell wall regeneration, in this study we investigated how cells sense ‘nakedness’ to rebuild their walls, probing a cell wall integrity monitoring system in *C*. *reinhardtii*.

The vegetative cell wall of dividing cells of *C*. *reinhardtii* is made almost entirely of proteins, including hydroxyproline (Hyp)-rich glycoproteins, and its multi-layered architecture makes it both hardy and flexible^9–11^. This architecture can accommodate a ten-fold increase in cell size during the light phase of the daily light/dark cycle. *C*. *reinhardtii* cells build a second type of cell wall during zygote development following the mating between *plus* and *minus* sexual gametes^12,13^. The mating reaction leads to the activation of a metalloprotease, gametolysin (g-lysin), which sheds the cell wall to allow gamete fusion and subsequent *de novo* assembly of a strong zygotic cell wall^8,14^. This zygotic wall is chemical-resistant and desiccation-tolerant, providing a safe environment for the zygotes to lay dormant until conditions are once again favorable^15–17^.

Of the cell wall structural components, many Hyp-rich glycoprotein-encoding genes are upregulated as early as 15 minutes after cell wall shedding by g-lysin treatment^18–20^. Hoffmann and Beck^21^ examined in detail the regulation of three gamete-specific (GAS) Hyp-rich pherophorin-encoding genes, *GAS28*, *GAS30*, and *GAS31*, whose expression increases upon g-lysin treatment without responding to variable osmotic conditions. This study suggested that cell wall removal upregulates *GAS* gene expression. It remains unknown how cell wall removal upregulates these three gamete-specific gene transcripts or whether their finding for these GAS genes is applicable to the other g-lysin-inducible cell wall-related genes.

The importance of signaling triggered by g-lysin treatment is suggested by the number of genes regulated by this signal. A recent study using transcriptome analysis revealed 143 genes up-regulated within one hour following g-lysin treatment^22^, suggesting that a signal triggered by g-lysin treatment may control the assembly of the vegetative cell wall. Comparative analysis of this g-lysin-induced transcriptome with an early zygote transcriptome identified two subsets of genes, distinguished by the presence or absence of upregulation in early zygotes^23^. The latter, the vegetative wall-specific g-lysin-induced gene subset (C24 or gL-EZ^23^) includes 36 Hyp-rich glycoprotein-encoding genes particularly enriched in the pherophorin family, likely specific for the vegetative wall structure. The other subset, which comprises genes common to both vegetative and zygotic walls (C44 or gL + EZ^23^), includes 67 genes involved in protein glycosylation and secretion, indicating that g-lysin-induced cell wall removal indeed controls cell wall assembly together with the upregulation of structural cell wall protein genes. Hereafter, we refer to these two subsets of cell wall-related genes as CW genes of the structural protein type and the protein processing type.

Here, we present mechanistic insights into the elusive signal generated by g-lysin-induced cell wall removal as a critical step forward from the pioneering study by Hoffmann and Beck^21^. First, we examined whether CW genes are activated via transcriptional and post-transcriptional mechanisms using our promoter-reporter transgenic strains. Second, we evaluated three signals: osmotic stress, the release of digested cell wall fragments, and the loss of cell wall integrity - expected during g-lysin treatment - as potential triggers for the activation of CW genes using cell wall defective (*cwd*) mutants that we isolated and reported in this study (see Supplementary Fig. S1 for illustrations of the three hypothetical signals).

Our data show that osmotic stress plays a critical role for fully activating cell wall-related gene expression, while compromised cell wall integrity plays a dominant role in this process regardless of g-lysin treatment. Taken together, we propose a new signaling mechanism that integrates osmosensing and the sensation of the mechanical integrity of cell walls in *C*. *reinhardtii*. Our g-lysin-inducible promoter-reporter system provides an excellent tool for a genetic screen where molecular components of cell wall integrity signaling can be identified.

## Results

### g-lysin treatment induces transcriptional activation of CW genes

A majority of the genes in the CW gene sets fell into two functional groups; protein processing (i.e., related to translation or glycosylation) and structural cell wall proteins. To survey the CW genes systematically, we selected three genes related to protein processing; *SEC61G*, *AraGT1*, and *RHM1*, and four structural cell wall proteins; *GAS28*, *GAS30*, *GAS31*, and *PHC19* (Table 1). According to the reads per million kilobases mapped (RPKM), taken from the Ning *et al*.^22^ dataset, which we used as a proxy for absolute expression level, the delta RPKM values between the g-lysin-untreated and g-lysin-treated cells for our selected genes indicate a substantial increase in gene expression within 1 hour upon g-lysin treatment (Table 1).

**Table 1:** Genes of interest selected from curated transcriptome data.

Name^1^	Locus ID^a^	Putative function^b^	ΔRPKM^c^	Promoter line
*PHC19*	Cre17.g696500	Cell wall pherophorin	594	yes
*GAS28*	Cre11.g481600	Cell wall HRGP	1351	no
*GAS30*	Cre11.g481750	Cell wall HRGP	73	no
*GAS31*	Cre11.g468359	Cell wall HRGP	2656	no
*SEC61G*	Cre16.g680230	ER protein translocase	1445	yes
*AraGT1*	Cre14.g629000	ER glycosyltransferase	80	yes
*RHM1*	Cre02.g083800	Rhamnose synthase	169	yes

^a^According to the Chlamydomonas annotation 5.6 (https://phytozome.jgi.doe.gov/).

^b^According to Joo *et al*.^23^.

^c^Difference in Reads Per Kilobase transcripts per Million reads (RPKM) between untreated and g-lysin-treated gametes, based on Ning *et al*.^22^.

**Figure 1:**
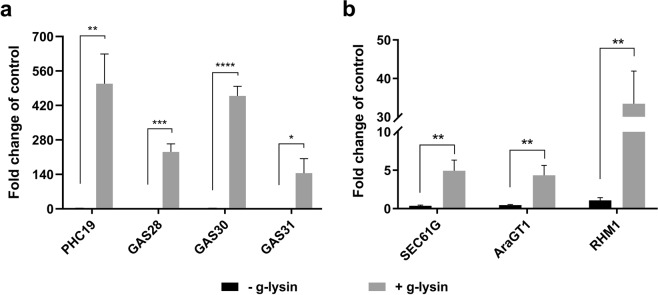
Transcription of CW genes in response to gametolysin treatment shows global upregulation. (**a**,**b**) Bar graphs represent the change in expression for cell wall protein-encoding (**a**) and protein processing-related (**b**) genes in wild type (CC-125) cells. Untreated control samples are represented by black bars, grey bars represent cells treated with g-lysin. Gene expression is quantified in terms of fold change compared to the samples before the g-lysin treatment. Error bars represent one standard deviation from the mean of biological triplicate samples. Welch’s t-test indicates statistical significance at p ≤ 0.05 (*), p ≤ 0.01 (**), p ≤ 0.001 (***), p ≤ 0.0001 (****); α = 0.05.

To confirm the reported transcriptome results and quantify the g-lysin induced gene expression, we analyzed the expression of our selected genes by reverse transcription and quantitative PCR (RT-qPCR) in gamete cells where little growth-related cell wall remodeling is expected. Following the treatment of gametic cells with g-lysin, a significant change in expression was observed for the structural protein genes, ranging between 145- and 508-fold increases (Fig. 1a). The protein processing genes showed modest increases between four- and 33-fold (Fig. 1b).

**Figure 2:**
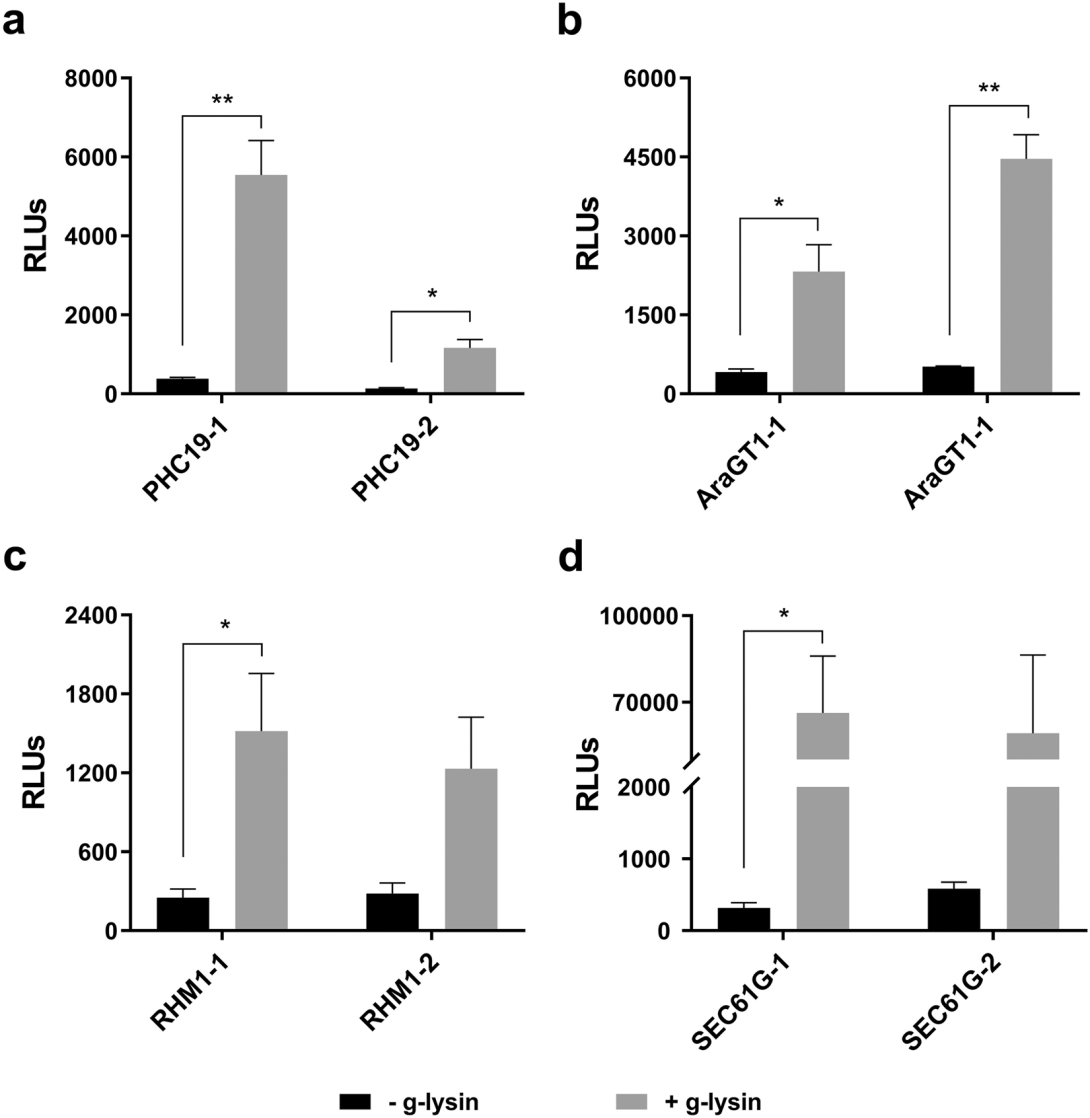
Promoter-driven luciferase activity increases in response to g-lysin treatment. Bar graphs represent the change in promoter activity of *PHC19* (**a**), *AraGT1* (**b**), *RHM1* (**c**), and *SEC61G* (**d**) genes, using two independent promoter-transformed lines. Luciferase activity is expressed as relative light units (RLUs) based on luminescence quantification. Untreated gamete control samples are represented by black bars. Grey bars represent gamete cells treated with g-lysin. Error bars are one standard deviation from the mean of biological triplicate data. Welch’s t-test indicates statistical significance at p ≤ 0.05 (*), p ≤ 0.01 (**); α = 0.05. RHM1-2 and SEC61G-2 showed a large variation that resulted in significance at 0.055 and 0.064, respectively, below the 95% cutoff.

To distinguish the transcriptional and the post-transcriptional mechanisms for this upregulation by g-lysin treatment, transgenic strains harboring promoter-luciferase constructs were used to probe promoter activities for *SEC61G*, *AraGT1*, *RHM1*, and *PHC19* genes, as described in our previous study^23^. Two independent transgenic lines for each promoter-reporter construct showed a definite increase with variation, ranging between 4- and 210-fold in luciferase expression in response to g-lysin treatment (Fig. 2). All four promoters tested displayed significant activation following g-lysin treatment in at least one of the two transgenic lines. This result suggests that many of the cell wall-regulated genes are activated at the transcriptional level. Note that the increase of promoter activity for a given gene did not always match the extent to which the transcript accumulated in response to g-lysin. For example, the *SEC61G* promoter showed a hundreds-fold increase in activity when treated with g-lysin, while the mRNA levels increased only modestly (Fig. 1). This discrepancy may be due to the post-transcriptional regulation of *SEC61G* expression.

### Translational inhibition revealed the complex regulatory network of the g-lysin-induced CW gene expression

**Figure 3:**
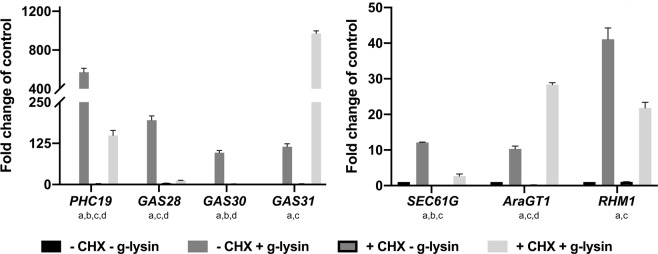
Transcripts are differentially affected by CHX pretreatment prior to gametolysin treatment. (**a**,**b**) Bar graphs represent the change in gene expression for cell wall protein (**a**) and protein processing type (**b**) CW gene transcripts in wild type (CC-125) cells. Untreated gamete control samples represented by black bars, −CHX −g-lysin; medium grey bars for CHX treated cells, +CHX; dark grey bars for cells treated with g-lysin, +g-lysin; light grey bars for both CHX and g-lysin-treated cells, +CHX +g-lysin. Gene expression is quantified in terms of fold change of the untreated control condition. Error bars represent one standard deviation from the mean of biological triplicate samples. Letters below gene names indicate a significant difference between the specified samples using a two-way ANOVA at p ≤ 0.01; α = 0.05; ‘a’ for −CHX −g-lysin *vs*. +g-lysin, ‘b’ for −CHX -g-lysin *vs*. +CHX, ‘c’ for −CHX -g-lysin *vs*. +CHX +g-lysin, and ‘d’ for +g-lysin *vs*. +CHX +g-lysin.

One of the critical features of gene regulatory networks is a hierarchical structure, where a primary response leads to a secondary response, dependent on the protein produced from the primary response. To examine such a hierarchy, we examined g-lysin-induced transcript accumulation in cells treated with the eukaryotic protein synthesis inhibitor cycloheximide (CHX). The CHX treatment itself minimally affected CW gene expression, showing no significant change across the tested transcripts (Fig. 3). By comparing the g-lysin-induced gene expression between CHX-treated and untreated cells, we identified three patterns. First, *RHM1* and *PHC19* showed significant reduction of the g-lysin-induced upregulation (two- and four-fold) but remained responsive to g-lysin treatment (Fig. 3). Second, *GAS28*, *GAS30*, *and SEC61G* showed near complete inhibition of the g-lysin-induced upregulation (86–97%) in CHX-treated cells, indicating that they are regulated by a secondary response, which is consistent with a previous report^17^. Third, *AraGT1* and *GAS31* showed three- and eight-fold increases in the g-lysin-induced upregulation when pretreated with CHX. This increase suggests that some of the early responsive CW genes such as *AraGT1* and *GAS31* are down-regulated as negative feedback by a regulator either short-lived or up-regulated by the g-lysin treatment. Overall, our result suggested that *AraGT1*, *RHM1*, *PHC19* and *GAS31* are primary targets and *GAS28*, *GAS30*, *and SEC61G* are secondary targets of the g-lysin-induced signaling pathway.

### Osmotic stress is necessary for full-scale CW gene activation

In *C*. *reinhardtii*, osmotic balance is controlled by the combined action of a pair of contractile vacuoles (CVs), which pump excess water out of the cell, and the cell wall, which protects the cell from lysing under the naturally hypotonic freshwater environments where *C*. *reinhardtii* live (Supplementary Text S1 for details). When cells lose their cell walls, osmotic stress is likely to be incurred on the cells. Cellular osmotic conditions can be monitored by using CV cycling as a proxy for water flux^24^.

To assess the effect of osmotic stress on CW gene expression, we analyzed cells transferred from Tris-acetate-phosphate (TAP), a standard growth medium (64 mOsm/L) to half-diluted TAP (1/2 TAP, 32 mOsm/L), where the CV cycling time shortens, and a sucrose-supplemented hypertonic condition (TAP + SS, 204 mOsm/L), where the CV cycle stops (Supplementary Table S1). The structural protein genes showed modest two- to four-fold upregulation when adjusted to the hypertonic condition (TAP + SS), whereas only non-significant changes were observed in the strong hypotonic condition (½ TAP) (Supplementary Fig. S2). *SEC61G* showed no significant difference under osmotic stress. Overall, the level of upregulation observed under osmotic stress is far smaller than the fold-induction following g-lysin treatment, suggesting only a minor role in CW gene regulation.

**Figure 4:**
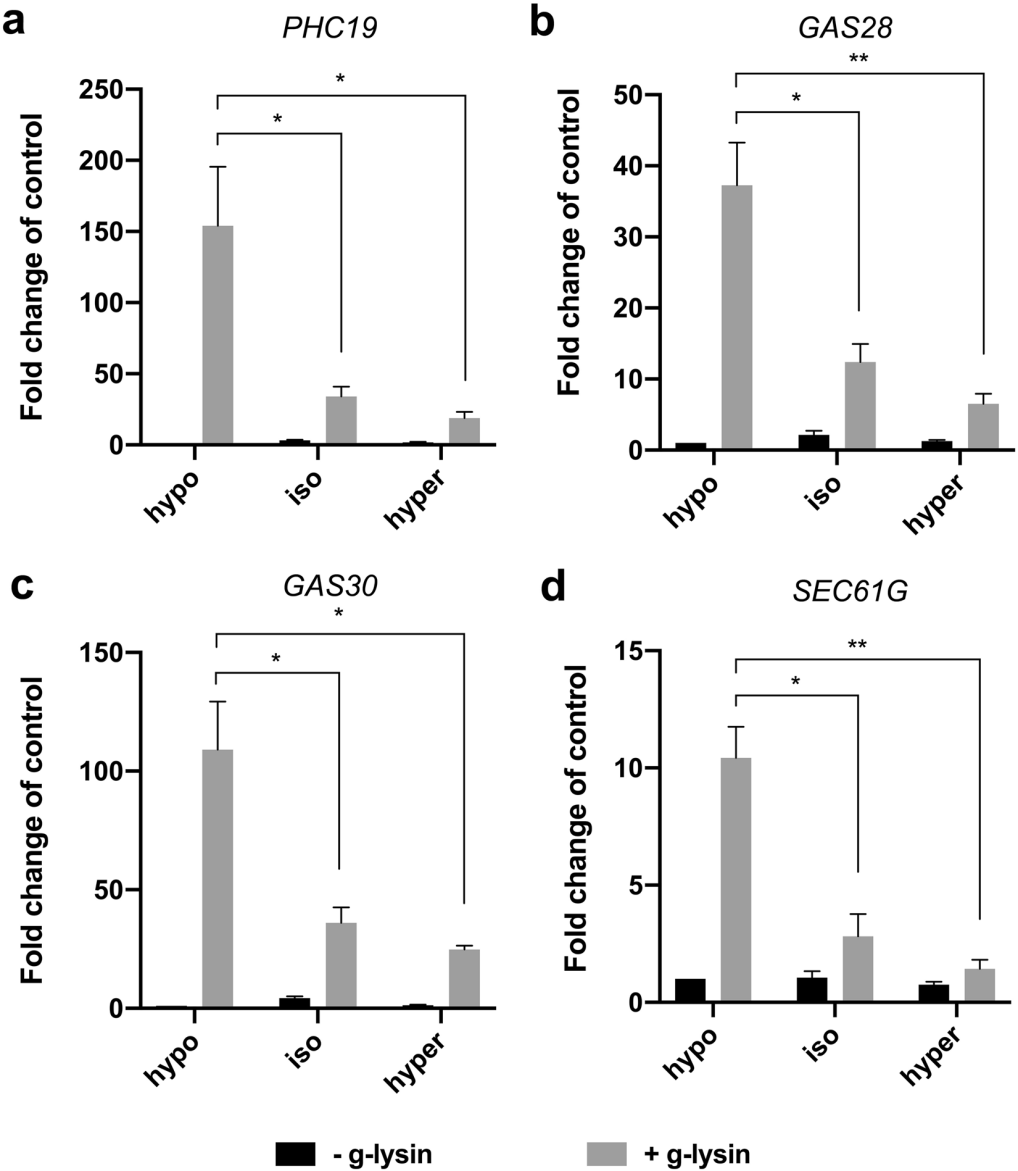
Isotonic and hypertonic conditions negatively affect the g-lysin-induced CW gene activation. (**a**–**d**) Bar graphs represent the change in gene expression by g-lysin treatment for *PHC19* (**a**), *GAS28* (**b**), *GAS30* (**c**), and *SEC61G* (**d**) in wild type (CC-125) cells. g-lysin treatment was done in three constant osmotic conditions, hypo- (64 mOsm), iso- (175 mOsm), and hyper- (204 mOsm). Untreated gamete control samples represented by black bars, −g-lysin; medium grey bars for g-lysin-treated cells, +g-lysin. Gene expression is quantified in terms of fold change of the untreated control in hypotonic condition. Error bars represent one standard deviation from the mean of biological triplicate samples. Welch’s t-test indicates statistical significance at p ≤ 0.01 (*), p ≤ 0.001 (**); α = 0.05.

It may be possible that osmotic stress becomes relevant for the cells to activate CW genes only if the cell wall is impaired or absent. To test this possibility, we designed an experiment that puts cells in different osmotic conditions while reducing osmotic stress to a minimum during g-lysin treatment. Cells were first adapted to hypo- (standard TAP, 64 mOsm/L), iso- (175 mOsm/L), and hypertonic (204 mOsm/L) conditions according to our CV cycle observations. The preconditioned cells were then treated with g-lysin prepared in media of the same osmolarity. In the iso- and hypertonic conditions, we observed a dramatic loss, between 67% and 89%, in the induction of CW genes by g-lysin treatment relative to the level in the hypotonic condition (Fig. 4). This result suggests that a hypotonic condition that gives rise to osmotic stress through the increase of turgor pressure or contractile vacuole cycling is critical for *C*. *reinhardtii* cells to fully activate the CW genes via the g-lysin-induced signaling pathway.

### Mechanical perturbation of the cell wall triggers the signal for CW gene activation

Our results discovered the critical importance of natural osmotic conditions for the full-scale activation of CW genes. Nonetheless, CW gene activation was not completely abolished even in the absence of contractile vacuole cycling, thus the trigger for CW gene activation remained to be determined. Thereby, we investigated the mechanical integrity of the cell wall as the potential trigger for CW gene activation. Cell wall integrity was examined by testing whether the cells lyse in the presence of 0.1% non-ionic detergent NP-40 (or Tergitol) – a substance to which cells with fully intact cell walls are undisturbed, while membranes of cells with defective cell walls are compromised. Earlier studies of cell wall-defective mutants that tested cells for lysis upon NP-40 treatment categorized mutant phenotypes into three distinct groups: (A) cells producing normal-looking walls attached to the plasma membrane, (B) cells producing walls but not connected to the plasma membrane, and (C) cells producing minute amounts of wall material^25–27^. These groups represent the three sequential consequences caused by g-lysin treatment: disruption of cell wall integrity, detachment of the wall from the plasma membrane, and complete removal of the wall. Therefore, whether CW genes are up-regulated without g-lysin treatment in cell wall defective mutants would inform about the involvement of cell wall integrity signaling for CW gene activation caused by g-lysin treatment.

**Figure 5:**
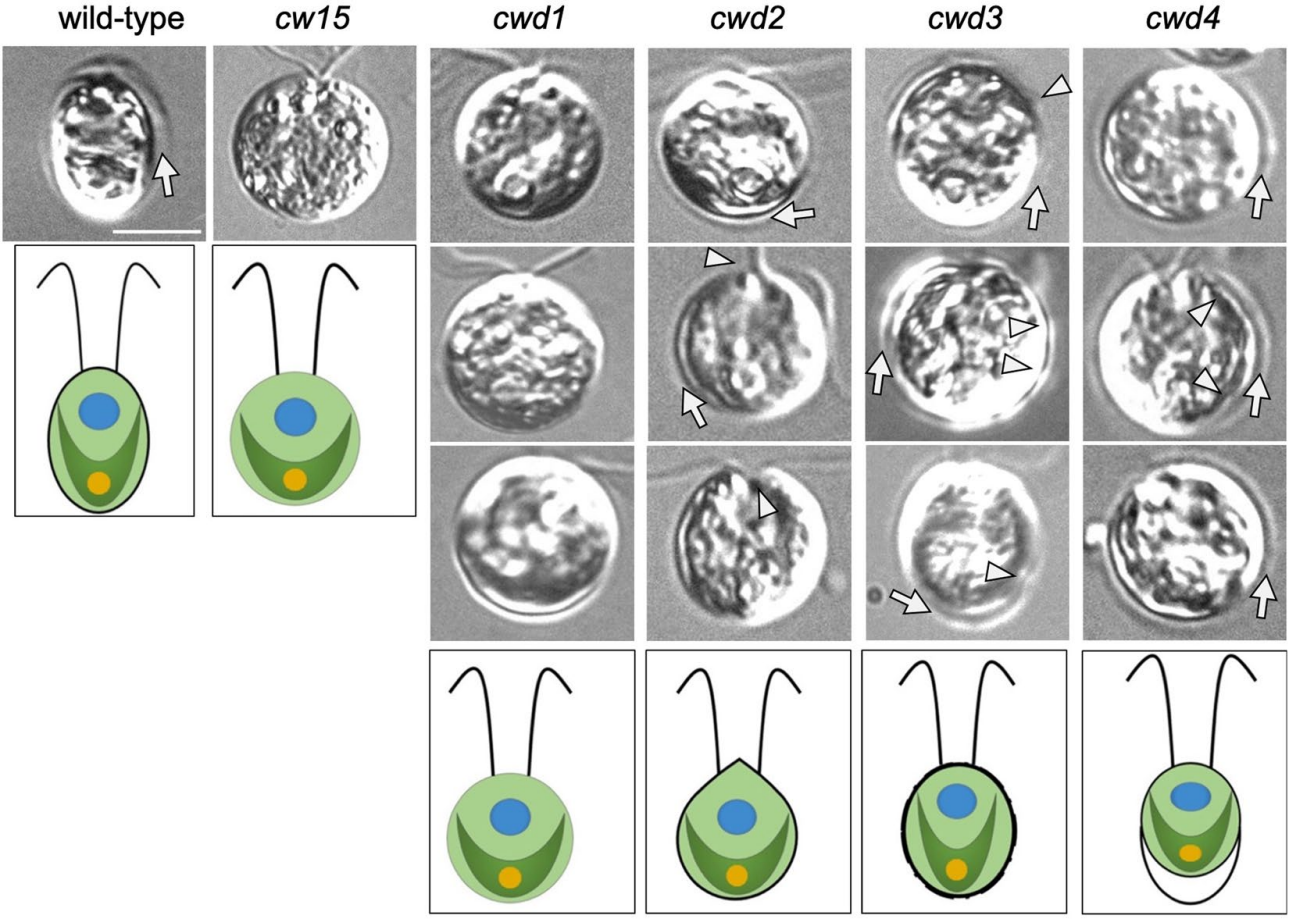
Phenotypic characteristics of wild-type and selected cell wall defective mutant lines. One to three representative micrographs per cell strain are given in columns (labeled above). Arrows indicate cell walls. Arrowheads point to strain-specific anomalies: *cwd2* with pointy or collapsed apical cell walls, *cwd3* with irregularity of its cell wall, and *cwd4* with detachment of the cell wall from the cell body. Scale bar = 5 μm. For each column, artistic renderings of wild-type and *cwd* mutant cells illustrating variations in cell wall or lack of cell wall are given at the bottom.

Most of the cell wall defective strains frequently used in *C*. *reinhardtii* research were isolated in the early 70s^25,26^. Therefore, the available cell wall defective strains may have accumulated spontaneous mutations that affect CW gene expression during their long-term culture. To assess the cell wall defective conditions without complex strain history, we isolated a new set of cell wall defective mutants based on their sensitivity to 0.1% NP-40. We selected a subset of these mutants based on NP-40 sensitivity and cell wall morphology to represent diverse mutant types. Our collection included four mutants (*cwd1-4*) from our mutant library and one historical mutant, *cw15*, whose phenotypes are summarized in Table 2. In our collection, *cwd1* and *cw15* contain no residual wall, based on their fully round shape and the absence of hollow in the phase-contrast images; *cwd2* and *cwd3* exhibit abnormal cell wall morphologies; and *cwd4* is a putative cell wall detachment mutant (Fig. 5). *cw15*, *cwd1*, *cwd3*, and *cwd4* showed high sensitivity to NP-40 (>90% cells burst within 2 min.) while *cwd2* showed medium sensitivity (70–90% cells burst).

**Table 2:** Summary of selected cell wall defective mutants.

Cell line	Cell morphology	Cell wall phenotype	cwd classification^a^	NP-40 sensitivity
*cw15* (CC-3491)	round cells	some material in medium	C	high
*cwd1*	round cells	no wall material present	C	high
*cwd2*	teardrop shaped; cell aggregates	unevenly distributed	B	medium
*cwd3*	oval cells	ruffled-looking edges	B	high
*cwd4*	gap between membrane and cell wall	normal looking	B	high

^a^(A) cells producing normal-looking walls attached to the plasma membrane, (B) cells producing walls but not connected to the plasma membrane, and (C) cells producing minute amounts of wall material, according to Davies and Plaskitt^25^.

**Figure 6:**
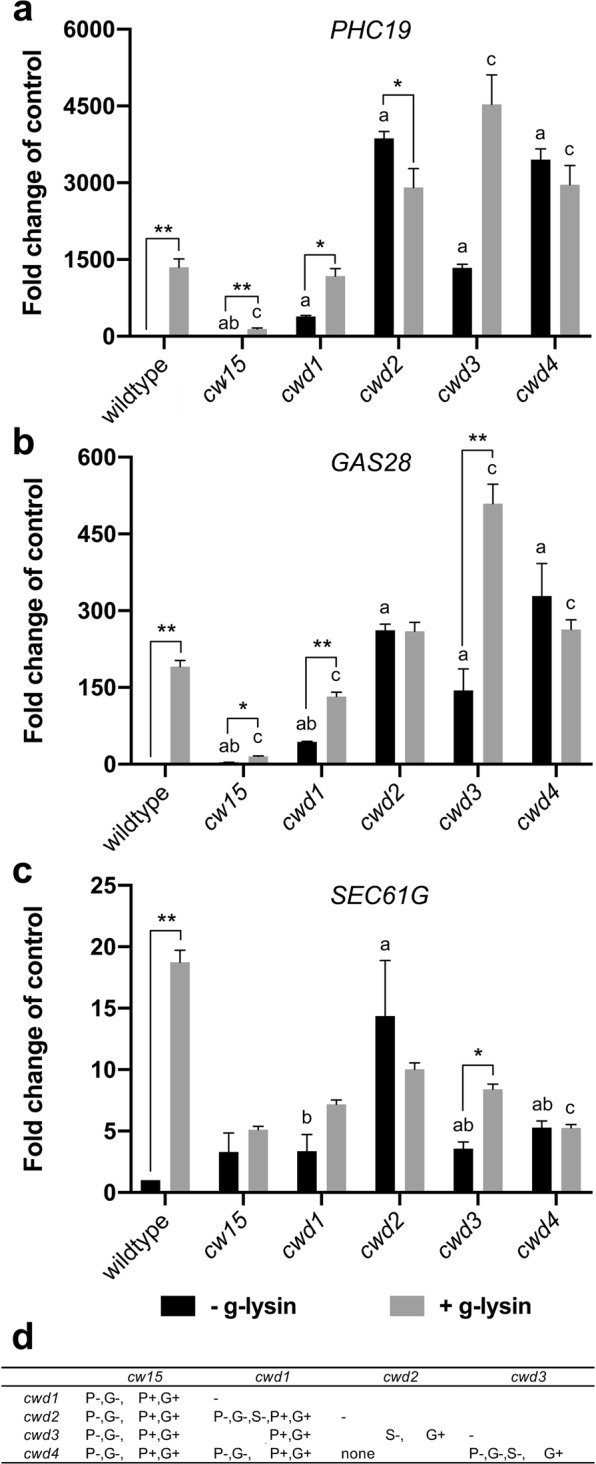
CW genes are constitutively activated in cell wall defective mutants. (**a**–**c**) Bar graphs represent the change in gene expression by g-lysin treatment for *PHC19* (**a**), *GAS28* (**b**), and *SEC61G* (**c**) in wildtype (CC-125), *cw15*, *cwd1*, *cwd2*, *cwd3*, and *cwd4*. Untreated gamete control samples represented by black bars, −g-lysin; medium grey bars for g-lysin-treated cells, +g-lysin. Gene expression is quantified in terms of fold change of the untreated wildtype cells. Error bars represent one standard deviation from the mean of biological triplicate samples. Asterisks indicate a significant difference between −g-lysin and +g-lysin cells using a two-way ANOVA at p ≤ 0.05 (*), p ≤ 0.01 (**); α = 0.05. Letters above error bars indicate a significant difference relative to the −g-lysin wild-type (‘a’) or the +g-lysin wild-type (‘b’ or ‘c’) cells using a two-way ANOVA (p ≤ 0.01; α = 0.05). (**d**) Statistical tests of difference in cell wall gene expression among the cwd mutants. Two-way ANOVA results (p ≤ 0.01; α = 0.05) between mutants given in the row and column headers are summarized for three cell wall genes (P, G, and S for *PHC19*, *GAS28*, and *SEC61G*) in -g-lysin cells (‘−’ sign) or in +g-lysin cells (‘+’ sign).

RT-qPCR analysis showed that all new *cwd* mutants expressed a much higher level of CW genes compared to the wild-type before g-lysin treatment (Fig. 6). When compared to the upregulated expression levels of the g-lysin-treated wild-type strain, untreated *cwd2*, *cwd3*, and *cwd4* cells exhibited comparable or higher expression of two structural CW genes, *PHC19* and *GAS28*, whereas untreated *cwd1* was found to show three to five times less expression of *PHC19* and *GAS28* than the other *cwd* mutants (Fig. 6a,b). *SEC61G* showed modest upregulation in all new *cwd* strains except *cwd2* but significantly less than the g-lysin-treated wild-type strain (Fig. 6c). *cwd2* and *cwd4*, which exhibited the highest expression of CW genes, showed no further upregulation following g-lysin treatment, suggesting that the g-lysin-induced signaling was fully activated before g-lysin treatment (Fig. 6). On the other hand, *cwd1* and *cwd3* with the more modest untreated CW gene expression showed, on average, 3.2-, 3.3- and 2.2-fold further upregulation of *PHC19*, *GAS28*, and *SEC61G* following the g-lysin treatment (Fig. 6). Since the prepared g-lysin likely contains cell wall debris as well as active g-lysin^28^, this residual response to g-lysin suggests that cell wall ligands that are absent or modified in *cwd1* and *cwd3* may be necessary for the full-activation of g-lysin-induced signaling. This gene expression analysis of new *cwd* mutants suggests the primary importance of cell wall integrity and the involvement of cell wall ligands-mediated signaling for CW gene regulation in *C*. *reinhardtii*.

An old mutant, *cw15*, however, showed an interesting exception. *PHC19* and *GAS28* were found to be expressed 19- and 3.6-fold higher in untreated *cw15* cells than in the wild-type, yet at least 20- and 12-fold lower than in *cwd1* – showing the lowest CW gene expression among the new *cwd* mutants (Fig. 6a,b). g-lysin treatment upregulated *PHC19* and *GAS28*, but at a modest level, less than ten-fold in *cw15*. We reasoned that the low CW gene expression of *cw15* even with g-lysin treatment might be due to its long-term adaptation to its cell wall-less condition where constant CW gene expression becomes wasteful. It is, therefore, conceivable that *cw15* may have accumulated a mutation preventing CW gene expression. To learn about whether such a suppressor mutation exists in *cw15*, sexual-recombinant progeny were generated by mating *cw15* with a cell wall-intact strain transformed with the *PHC19*-luciferase construct (PHC19-2 in Fig. 2). A consistent 2:2 segregation of the cell wall defect among the progeny indicates that a single *cw15* mutation is likely responsible for the cell wall defect in the recombinant progeny (data not shown). We selected one tetrad in which two progeny with the *PHC19* reporter are distinguished by their cell wall phenotypes: one with intact cell walls as a *CW15* strain and the other with defective cell walls as a *cw15* strain. Luciferase activity of the *CW15* progeny showed a 4.9-fold increase by the g-lysin treatment, whereas the *cw15* progeny showed constitutive reporter activity at the similarly high level of the g-lysin-treated *CW15* strain (Supplementary Table S2). Upregulated *PHC19* and *GAS28* gene expression ascertained the recovery of CW gene expression to a comparable level to *cwd1* in the selected *cw15* progeny (Supplementary Fig. S3). These results further support the importance of cell wall integrity, which when compromised, triggers the upregulation of CW genes in *C*. *reinhardtii*.

## Discussion

**Figure 7:**
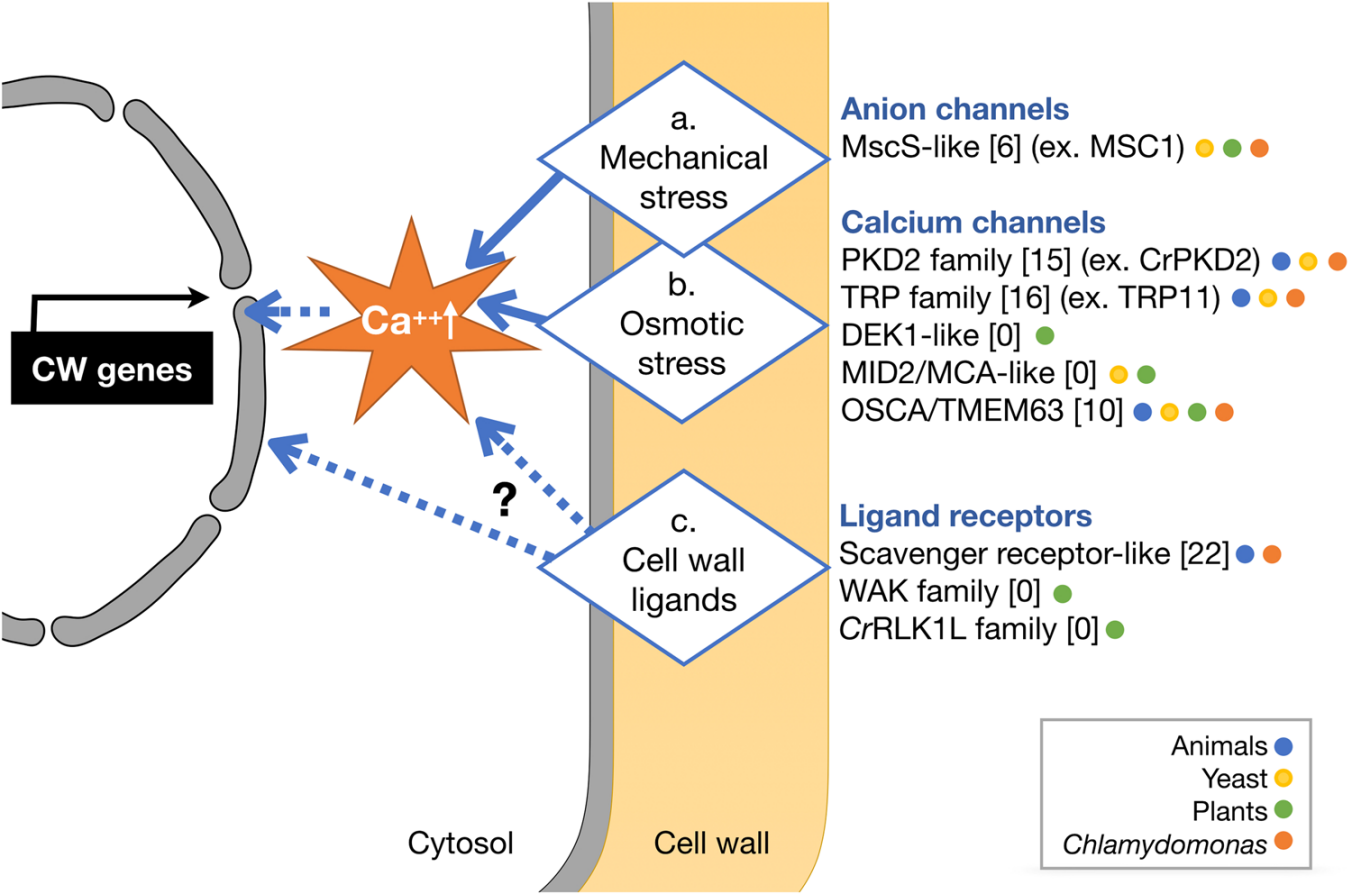
A model of cell wall integrity signaling in *C*. *reinhardtii*. This model illustrates three sensing mechanisms that may crosstalk via Ca^++^ oscillation and/or protein phosphorylation for rapid upregulation of CW genes upon cell removal by g-lysin treatment. Solid arrows indicate signaling mechanisms with experimental evidence. Dashed arrows depict putative signaling mechanisms. A sensory role of mechanical and osmotic stresses is so far reported for mechanosensitive channels in six different families (**a**,**b**). Three protein families of known or putative receptors sensing cell wall-associated ligands may be involved in CW gene regulation directly or indirectly via mechanosensitive channel-dependent signaling (**c**). Numbers in brackets indicate the number of candidate genes found in the *C*. *reinhardtii* genome. Functionally characterized proteins of *C*. *reinhardtii* are given as examples. Colored dots indicate which organisms possess genes for the given family. MscS, mechanosensitive channel of small conductance; PKD2, polycystic kidney disease 2; TRP, transient receptor potential; MCA, *mid1*-complementing activity; WAKs, wall-associated kinase; *Cr*RLK1L, *Catharanthus roseus* receptor-like kinase 1-like protein.

This study is focused on how cells regulate CW gene expression in response to ‘nakedness’ and rebuild their important wall. By analyzing CW genes activated by cell wall removal and imperfect cell walls, we propose a signaling mechanism that consists of three sensory modules that trigger, assess, and monitor cell wall assembly, whose details are explained below (summarized in Fig. 7).

For the primary trigger of CW gene expression, we propose a cell wall integrity signaling that senses mechanical defects of the cell wall (Fig. 7a; Supplementary Fig. S1c). Full-scale CW gene activation, similar to cells treated with g-lysin, was observed in *cwd2* and *cwd4*, - mutants whose cells are either enclosed or detached from the cell wall but still maintain contact with the wall - without g-lysin treatment (Fig. 6). This result suggests that a complete lack of cell wall is not required for CW gene activation and instead a compromised integrity of the cell wall is sufficient.

The cell wall is the outermost layer that plays a significant role in water balance by protecting cells from lysing in hypotonic conditions, like the freshwater environment (and natural habitat) of *C*. *reinhardtii*. It is expected that the removal of the cell wall would invoke immediate osmotic stress in hypotonic conditions since turgor pressure contained by the cell wall will be lost and this loss will be realized as stress on the plasma membrane (Supplementary Fig. S1a). When wild-type CC-125 cells were transferred to media of varying osmolarity, we observed little change in CW gene expression level (Supplementary Fig. S2). In contrast, we found a 67–89% reduction of g-lysin-induced CW gene activation when cells were preconditioned in iso- or hypertonic media (Fig. 4). In the iso- and hypertonic condition, contractile vacuole cycling was not observed, indicating that water influx was not significant. In the absence of water influx, cells may survive even if the cell wall is imperfect or absent. It is, therefore, logical that building an intact cell wall may become less critical under iso- and hypertonic conditions. We thereby suggest that osmosensing invoked upon cell wall removal serves as a precondition for rapid CW gene activation but is not the primary trigger (Fig. 7b).

What can be the advantages of sensing both mechanical stress and osmotic condition? Osmosensing may help to modulate cell wall assembly according to cellular need. Cell growth and associated cell wall assembly may proceed as normal in the presence of enough turgor, allowing for cell wall expansion, whereas full-scale and rapid cell wall assembly can be activated if cells are in danger of bursting upon damage or loss of the cell wall. Osmosensing has also been noted as a critical factor for the activation of genes involved in cellulose and carbohydrate metabolism by mechanical perturbation of cell walls in plants^29^.

Two naked strains, *cw15* and *cwd1*, showed <5–30% of the fully activated level of CW gene expression before g-lysin treatment and further upregulation following g-lysin treatment (Fig. 6). These results elucidate critical aspects of the signaling induced by g-lysin treatment. First, cells lacking a normal cell wall continuously attempt to rebuild their cell wall as indicated by the constitutive activation of CW genes. Secondly, naked cells partially induce CW gene activation and are still able to respond to g-lysin lysates containing digested cell wall material. The latter offers a hypothesis that the activation of CW genes may utilize biochemical signaling triggered by the perception of cell wall-derived ligands (Supplementary Fig. S1b). This hypothesis may be tested by comparing the effects of various gametolysin preparations following heat denaturation, treatment with sugar-cleaving enzymes, and size fractionation, which will elucidate the biochemical nature of said elusive ligands. Such biochemical signaling may be utilized for the induction of localized cell wall synthesis when sensing atypical wall structures exposed by wounding or pathogen invasion.

Which mechanosensitive proteins on the cell surface may trigger CW gene activation? In *C*. *reinhardtii*, mechanosensitive channel activities have been detected on the flagella and cell body^30,31^ and three types of these channels have so far been characterized: TRP11, regulating flagellar beating in response to mechanical bumping^32^; CrPKD2, mediating signal transduction induced by flagellar agglutination during mating^33^; and MSC1, localized to the chloroplast envelope^34^. Although these channels are not on the plasma membrane where the cell wall sensors are presumably localized, similar mechanosensitive channels associated with the cell wall may play a sensory role for CW gene activation.

Cell wall integrity signaling has been extensively studied in yeast and plants^4–6^. In *Saccharomyces cerevisiae*, Wsc1 and Mid2 serve as the primary sensors of yeast cell wall integrity, both of which are single-transmembrane domain proteins with an *O*-mannosylated Ser/Thr-rich extracellular domain that stretches like a nano-scale ‘spring’ in response to mechanical stresses on the cell wall^35,36^. In *C*. *reinhardtii*, many of the surface receptor/channel proteins in the TRP (transient receptor potential) family contain one or more extracellular hydroxyproline-rich domains that are presumably *O*-glycosylated, which may transduce mechanical stresses of the cell wall in an analogous manner to yeast Wsc1 and Mid2 (Supplementary Table S3). In plants, two mechanosensitive calcium channels are described: DEK1, critical for epidermal cell differentiation and adhesion^37,38^ and Mid1-complementing MCA1, a plant-specific calcium channel necessary for touch and cold sensing in the roots^39,40^. Recently, it was shown that MCA1 mediates cell wall damage responses in *Arabidopsis*^41^. DEK1 homologs are found in several protozoan genomes (J.-H. Lee, unpublished), and whether DEK1 is involved in cell wall integrity signaling remains to be studied.

Osmotic stress responses have been studied in *C*. *reinhardtii*. Previous genetic studies described five mutants (*osm* and *osmo*) defective in mitigating osmotic stresses (*cw15* background)^42,43^; however, their genetic mutations were not reported, with the exception of OSMO75, a SEC6 homolog involved in CV cycling^43^. Recently, calcium-influx invoked by osmotic stress has been characterized, suggesting that osmosensing in *C*. *reinhardtii* employs calcium-dependent signaling^44^. In a wide range of organisms, osmotic stress invokes a rapid calcium influx, which is mediated by mechanosensitive channel proteins in the TRP family that can sense a variety of stimuli^45^. In plants, no TRP family genes are found. Recently, OSCA1 was identified as a calcium channel gated by osmotic stress in *Arabidopsis*^46^. OSCA1 and its homologs in animals, known as TMEM63, belong to the DUF221-containing protein family that is conserved throughout eukaryotes^47,48^. The *C*. *reinhardtii* genome contains a large number of TRP family channels and 10 OSCA1-like channels, offering potential candidates involved in cell wall integrity signaling (Supplementary Table S3).

How do the cell wall integrity and osmotic stress pathways interact? The majority of eukaryotic mechanosensitive and osmosensing channels, with the exception of the MscS family, are calcium channels and their crosstalk is predictable. Another type of crosstalk may exist between downstream signal transduction pathways. In *S*. *cerevisiae*, Mid2, a cell wall integrity sensor, interacts with a small G-protein Rho1 for downstream signaling involving Pkc1 and the MAP kinase cascade^4^. Interestingly, osmotic stress sensed by Mid1, a stretch-gated calcium channel, and Mid2-Rho1-Pkc1-mediated signaling share Skn7, a two-component response regulator, as a downstream transcriptional regulator^49^. In *Schizosaccharomyces pombe*, a PKD2-like channel is complexed with Rho1 and transduces mechanical stress, leading to Rho1 activation that regulates cell wall synthesis^50^.

Cell wall ligand-mediated signaling is extensively studied in plants, which utilizes receptor-like protein kinases of two different families^5,51^. The first is the *Catharanthus roseus* receptor-like kinase 1-like (*Cr*RLK1L) family such as THESEUS1 and FERONIA^52^. *Cr*RLK1Ls have an extracellular domain containing one or two malectin-homology domains known to bind to disaccharides such as nigerose or maltose (Glc-a1-3-Glc or Glc-a1,4-Glc)^53^, whose signaling is understood to regulate various plant wall features such as stiffening^54^. Recent biochemical and structural studies of the extracellular domain of FERONIA have shown its binding to a peptide ligand, RALF1 (rapid alkalinisation factor1), and its 3D-structure reveals significant difference from previously characterized malectin-homology domain, suggesting its binding to disaccharides less likely^55,56^. The extracellular domain of FERONIA also binds to LRR domain of LRX4 (leucine-rich repeat extensin4) that includes a hydroxyproline-rich extensin domain associated with the cell wall matrix, providing a mechanical link of *Cr*RLK1Ls to the cell wall^57^. The second is the wall-associated kinase (WAK) family with an extracellular domain (Pfam:13947) that are shown to bind pectin-derived oligo-galacturonides (OGs) and pectins^58–60^. WAKs are critical for controlling plant cell expansion, possibly via their interaction with GRPs (glycine-rich proteins)^61,62^. BRI-dependent brassinosteroid signaling is another critical component of cell wall integrity signaling, which leads to the activation of cell wall-loosening genes via hormonal cues^63^.

Although no homologs to *Cr*RLK1Ls, WAKs, or BR-signaling components are found in *C*. *reinhardtii*, surface receptors localized in the plasma membrane have been reported, including the sex-agglutinins that mediate gamete recognition during mating^64,65^ and the elusive receptor for the sex-inducing pheromone studied in *Volvox carteri*, a close relative of *C*. *reinhardtii*^66,67^. The *C*. *reinhardtii* genome encodes >20 scavenger-receptors cysteine-rich (SRCR)-domain proteins that are known to perceive endogenous and foreign ligands at the cell surface in animals^68,69^. We found that SRCR proteins in *C*. *reinhardtii* possess various lectin domains and putative hydroxyproline-rich domains for their cell wall association, and five of which are TRP-type channels, suggesting their potential roles in calcium-mediated signaling (Supplementary Table S3).

This study evaluated three putative signaling pathways that are involved in regulating CW genes and, presumably, the cell wall assembly of *C*. *reinhardtii*. To complement our study using cell wall defective mutants, an investigation of available osmosensing mutants for g-lysin-induced gene expression and cell wall regeneration will likely refine our proposed model of cell wall integrity signaling in *C*. *reinhardtii*. A plausible approach to identify the molecular components of cell wall integrity signaling in *C*. *reinhardtii* is a forward genetics screen for mutations that abolish constitutive activation of CW genes in cell wall defective mutants. Future studies combining such forward and reverse genetics approaches will hopefully uncover the molecular details of this cell wall integrity signaling in *C*. *reinhardtii* and invite comparative studies that ask whether cell wall signaling is conserved between plants and *C*. *reinhardtii* despite their distinct cell wall composition and organization.

## Methods

### Strains and culture conditions

*C*. *reinhardtii* strains, CC-125 (wild-type, *mt*+), CC-621 (*mt*−), CC-2663 (*nic7*; *mt*−) and CC-3491 (*cw15*, *mt*−) were obtained from the Chlamydomonas Resource Center (www.chlamydollection.org). All *C*. *reinhardtii* strains were maintained and cultured under medium light (50 μmol photons m^−2^ s^−1^) at 23 °C in Tris-acetate-phosphate (TAP) medium^7^ solidified with 1.5% Bacto agar. *cwd1*, *cwd2*, *cwd3*, *and cwd4* were isolated from mutagenized population of JL28 (*nic7*; *mt*−) that was generated by mating between CC-125 and CC-2663 (*nic7*; *mt*−). Nitrogen-free TAP medium was prepared by omitting nitrogen from TAP medium. Liquid media with varying osmolarities were made by either adding amounts of sucrose to liquid TAP media or by diluting the media with water. ½ TAP, 1:1 water to media; TAP + S, 60 mM sucrose; TAP + SS, 120 mM sucrose.

### Gametogenesis

Seven-day-old cells grown on TAP plates were harvested and suspended in nitrogen-free TAP (NF-TAP), then counted and normalized to a concentration of 5 × 10^7^ cells/mL. Suspended cells were incubated under high light (200 μmol photons m^−2^ s^−1^) for a minimum of 3 hours to induce gametogenesis. Sufficient gametogenesis was determined by a mating efficiency analysis as per the method described in Hoffman and Goodenough^70^, with 80% mating efficiency being the acceptable lower limit before continuing the experiment.

### Isolation of cell wall defective mutants by insertional mutagenesis

To isolate cell wall defective mutants, we followed the mutant screen using the method described by Davies and Plaskitt^25^. Abnormal colony morphology was screened by scanning plate cultures on TAP medium solidified with 2% Bacto agar under a dissecting microscope (S8APO, Leica) in a mutant pool generated by insertional mutagenesis. Putative mutant colonies were resuspended in liquid TAP medium and tested for the NP-40 sensitivity (detailed in the methods section). Mutants displaying >50% NP-40 sensitivity were selected as cell wall defective (*cwd*). Insertional mutagenesis was performed by the glass bead-assisted transformation using nicotinamide-requiring mutant cells (*nic7*, mt-) as described^71^. The plasmid used in the insertional mutagenesis was prepared by adding pHsp70A/RbcS2-AphVIII^72^ in pNic7.9^73^. The plasmid was linearized by *Eco*RI that cleaves between the pHsp70/RbsC2 promoter and the open reading frame of the AphVIII.

### g-lysin and CHX treatment

g-lysin extract was prepared as described^17^. For testing osmosensing, g-lysin was prepared from sexually competent gametes that were preconditioned in hypo-, iso-, and hypertonic conditions before mixing. Prepared g-lysin extract was then frozen at −80 °C until use. For g-lysin treatment, suspended cells were mixed with an equal volume of thawed g-lysin extract for 1 hour to ensure cell wall removal. g-lysin efficiency was determined by NP40 sensitivity test (see “NP40 sensitivity testing” in Materials and methods). Untreated control samples were mixed with an equal volume medium to maintain equal cell concentrations across samples. Cells were then incubated for 1 hour before harvesting RNA.

For CHX pretreatment, suspended cells were mixed with a small volume of 10 mg per mL CHX stock to a final concentration of 10 µg/mL, then incubated for 45 minutes. Following the pretreatment, some cells were treated further with an equal volume of thawed g-lysin extract. Cells were then incubated for one hour before harvesting RNA.

### Reverse transcription and quantitative PCR (RT-qPCR)

Total RNA extraction, cDNA synthesis, and qPCR reactions were carried out essentially as described^23^. In brief, each qPCR run had technical duplicate samples to generate average quantification cycle (Cq) data per run. Relative expression levels in each cDNA sample were normalized to the *RACK1* reference gene under the same conditions. Relative expression was calculated by the method described in Pfaffl^74^, which accounts for difference in PCR primer efficiency for the different transcript targets. Three biological replicates were averaged for quantitative analysis. Welch’s t-test was applied to the analyzed expression data or ANOVA was applied to the log-scale qPCR data (delta Cq) to check for statistical significance between treatment conditions. Primer sequences are presented in Supplementary Table S4.

### Luciferase activity assays

To induce promoter-reporter activity, promoter-luciferase-transformed lines were subjected to treatment conditions. Secreted Gaussian luciferase enzyme (from *Gaussia princeps*, codon-optimized for C. *reinhardtii*^75^) was collected from samples with equal cell concentrations by aliquoting 100 μL of sample into 1.5 mL centrifuge tubes and centrifuging for 3 minutes at 6,000 g. 40 μL of supernatant was then transferred from each sample to PCR strip tubes and mixed with 10 μL *Renilla* luciferase lysis buffer (Promega). Prepared samples were then stored at −20 °C until luciferase assays were run. Samples for luciferase assay were prepared by mixing 25 μL luciferase assay reagent (luciferase assay buffer + 1x final concentration luciferase assay substrate) (Promega) with 5 μL of thawed samples in a 384-well microtiter plate. The relative amount of luciferase enzyme in each sample was then quantified by the amount of luminescence detected by a BioTek Synergy 2 microplate reader.

### NP-40 sensitivity testing

20 μL of suspended cells were mixed with 20 μL 0.2% NP-40 “Tergitol” detergent (Sigma) in a 1.5 mL tube. Another 20 μL of suspended cells were simply diluted with media as an untreated control. 10 μL of mixture and 10 μL of untreated cells were loaded onto either side of a hemocytometer and visualized using brightfield microscopy with 100× total magnification. Both treated and untreated cells were counted to compare cell concentrations and determine the number of cells that had burst from the NP-40 treatment. This detergent disrupts the lipid-based membrane and will therefore cause any cells without an intact cell wall to lyse. A simple ‘NP-40 sensitivity’ value was then calculated by dividing the untreated cell concentration by the treated cell concentration and multiplying by 100%. For example, if all cells in a sample treated with NP-40 burst, the NP-40 sensitivity would be 100%.

### Contractile vacuole visualization and timing

To ascertain osmotic conditions of the media used in experiments, we measured the CV cycle (contractile vacuole cycling times) in the wild-type and a cell wall-less strain, *cw15*, under various osmotic conditions as defined in Komsic-Buchmann *et al*.^43^, either hypotonic media, half-diluted standard medium TAP (½ TAP, 32 mOsm/L) and the standard TAP (64 mOsm/L), or hypertonic media, the TAP supplemented with sucrose (TAP + S, 144 mOsm/L; TAP + SS, 204 mOsm/L) and incubated for an hour. 8 μL of cell suspension was loaded onto glass slides and cells were viewed under 1000× total magnification using Zeiss Axioscope A1. The CV cycle was measured manually by timing consecutive systole and diastole cycles of a single contractile vacuole per cell. A minimum of three cells per cell line per osmotic condition were measured.

We confirmed that increasing osmolarity in the media extended the CV cycles, indicating slow water influx. This trend was found in all the cell lines tested (Supplementary Table S1). The CV cycles were observed in the TAP + S condition but none in the TAP + SS condition, indicating that cells have their osmolarity between 144 and 204 mOsm per L, which agrees with the published cytosolic osmolarity of ~171 mOsm per L^24^. The *cw15* cells showed much longer contractile vacuole cycle times, nearly double the length of the wild-type cycles. In pure water (0 mOsm/L), the wild-type cells showed a contractile vacuole cycle time at the average of 9.56 s, whereas the *cw15* cells exhibited an average time of 17.66 s. The correlation between the cell surface area and the CV cycle was previously noted^43^.

## Supplementary information

Supplementary Text, Tables and Figures
