# Supplementary material for: Cell wall integrity signaling regulates cell wall-related gene expression in Chlamydomonas reinhardtii
Source: Sci Rep. 2019 Aug 21;9:12204. doi: 10.1038/s41598-019-48523-4 (PMC6704257; doi:10.1038/s41598-019-48523-4)

Supplementary information for Cronmiller et al. (2019) "Cell wall integrity signaling regulates cell wall-related gene expression in *Chlamydomonas reinhardtii",* submitted to Scientific Reports.

Evan Cronmiller, Deepak Toor, Nai Chun Shao, Thamali Kariyawasam, Ming Hsiu Wang, and Jae-Hyeok Lee

Corresponding Author:
Jae-Hyeok Lee
6270 University Blvd., Vancouver, British Columbia, V6T 1Z4, Canada
jae-hyeok.lee@botany.ubc.ca

Supplementary Text S1. Osmoregulation in *Chlamydomonas reinhardtii.*
Supplementary Table S1. The CV period (contractile vacuole cycling times) in five different media of varying osmotic conditions.
Supplementary Table S2. *PHC19* promoter-driven luciferase activity of cw15 progeny in response to g-lysin treatment.
Supplementary Table S3. Candidate genes for putative cell surface sensors involved in the cell wall integrity signaling of *C. reinhardtii*.
Supplementary Table S4. Primers used in this study**.**
Supplementary Figure S1. Hypothetical signaling mechanisms for the g-lysin-induced responses.
Supplementary Figure S2. Transcript expression suggests modest cellular response to changing osmotic environment.
Supplementary Figure S3. *cw15* progeny showed constitutive CW gene expression.
Supplementary Figure S4. Diagrammatic representation of osmoregulation in *C. reinhardtii* cells.

Supplementary Text S1. Osmoregulation in *Chlamydomonas reinhardtii*

As *C. reinhardtii* is primarily a soil and freshwater-dwelling organism, tight osmoregulation is necessary for survival and to carry out cellular processes. This regulation is maintained by a number of mechanisms, most notably the pumping of water out of the cell via the contractile vacuoles (CVs) and the selective uptake of solutes from the environment. In higher plants, turgor pressure generated in the periplasmic space between the plasma membrane and cell wall is also known to contribute to osmoregulation. Whether turgor pressure plays a role in osmoregulation in *C. reinhardtii* is unclear, as early researchers assume yes (Hoffmann & Beck, 2005) and others assume no when considering the flexibility of the cell wall (Komsic-Buchmann et al., 2014). The model presented in Supplementary Figure S4a is an illustration demonstrating how *C. reinhardtii* cells are likely to maintain a suitable intracellular osmolarity. In a hypotonic environment, water will tend to move into the cell via osmosis. Anytime the intracellular water volume is too high (solute concentration is too low), the contractile vacuoles will pump the excess out of the cell. In a hypertonic environment, it is hypothesized that cells will pump solutes into the cell and/or accumulate solutes in the cytosol to avoid water loss, but direct evidence for this mechanism is lacking.

A measurement of the osmotic balance in these cells is known as the water potential – the tendency of water to enter or leave a cell – and can be understood as the sum of many pressure potentials. The pressures exerted on cells include: solute potential (Ψ_s), pressure potential (Ψ_p), gravimetric potential (Ψ_g), and matrix potential (Ψ_m) (Taiz et al., 2015). In an aquatic environment, gravimetric and matrix potentials are negligible, so pressure potential and solute potential are crucial to aquatic organisms (Taiz et al., 2015). It remains unclear how much of an effect the pressure potential has on *C. reinhardtii.* Certainly, the aqueous environment will exert a certain amount of hydrostatic pressure on the cell, but whether a turgor pressure is generated between the plasma membrane and cell wall is unknown. Given the specific water potential of a *C. reinhardtii* cell, the cell's surface area, and the permeability of water across the plasma membrane, one can generate an understanding about the rate of water movement into or out of the cell, called water flux (see equation in Supplementary Figure S4b).

A limited number of studies have been published about osmoregulation in *C. reinhardtii*. Existing research has focused primarily on the organization and mechanism of the CVs and their role in water efflux. Early studies by Luykx et al. (1997) demonstrated CV formation to be the dynamic amalgamation of scores of smaller water-containing vesicles, which eventually fuse with the plasma membrane as a large contractile vacuole and force water out of the cell. They also found several osmoregulatory mutants to have contractile vacuole defects, who's poor viability confirmed the importance of the CV in maintaining cytosolic osmolarity (Luykx et al., 1997b). More recent studies have found and characterized specific components of the CV system, such as the vesicular membrane protein SEC6 that facilitates CV membrane fusion and the aquaporin MIP1 which pumps water from the cytosol into the CV vesicles (Komsic-Buchmann et al., 2012; Komsic-Buchmann et al., 2014).

A study by Hoffmann & Beck (2005) considered the effects of cell wall removal on osmoregulation using three previously identified transcripts *GAS28, GAS30,* and *GAS31.* Their results indicated that in both hypo- and hyperosmotic media, cells will accumulate high levels of all three transcripts within two hours. However, this study may contain inaccuracies as the reported osmolarities for the test conditions and cytosol of *C. reinhardtii* differ greatly from the more recently calculated values presented in Komsic-Buchmann et al. (2014).

This limited research has focused almost solely on the cell's response to hypotonic environments and how cells actively remove excess water to maintain a water balance. We know very little about the cellular responses to hypertonic environments, aside from the cessation of contractile vacuole activity and decreased expression of *SEC6* and *MIP1* (Komsic-Buchmann et al., 2012, 2014). One study observed the intracellular accumulation of glycerol in cells exposed to a hypertonic environment (León and Galván, 1995). This response was presumed to prevent water loss in cells by raising the intracellular solute content.

References

Hoffmann, X. K., & Beck, C. F. (2005). Mating-induced shedding of cell walls, removal of walls from vegetative cells, and osmotic stress induce presumed cell wall genes in *Chlamydomonas*. *Plant physiology*, *139*(2), 999-1014.

Komsic-Buchmann, K., Stephan, L. M., & Becker, B. (2012). The SEC6 protein is required for contractile vacuole function in *Chlamydomonas reinhardtii*. *J Cell Sci*, *125*(12), 2885-2895.

Komsic-Buchmann, K., Wöstehoff, L., & Becker, B. (2014). The contractile vacuole as a key regulator of cellular water flow in *Chlamydomonas reinhardtii*. *Eukaryotic cell*, *13*(11), 1421-1430.

León, R., & Galván, F. (1995). Metabolic pathway for glycerol synthesis under osmotic stress in the freshwater green alga *Chlamydomonas reinhardtii*. *Plant physiology and biochemistry*, *33*(2), 213-218.

Luykx, P., Hoppenrath, M., & Robinson, D. G. (1997). Structure and behavior of contractile vacuoles in *Chlamydomonas reinhardtii*. *Protoplasma*, *198*(1-2), 73-84.

Taiz, L., Zeiger, E., Møller, I. M., & Murphy, A. (2015). *Plant physiology and development*. Sinauer Associates, Incorporated.

Supplementary Table S1. The CV period (contractile vacuole cycling times) in five different media of varying osmotic conditions. Data is presented in second. n = 3. NCV indicates no contractile vacuole cycle**.**

Cell line	Media condition (osmolarity)				
	H_2O (0 mOsm)	½ TAP (32 mOsm)	TAP (64 mOsm)	TAP+S (144 mOsm)	TAP+SS (204 mOsm)
CC-125	9.56 ± .40	10.90 ± .50	16.64 ± 1.12	22.91 ± 1.28	NCV
CC-3491	17.66 ± 1.90	21.24 ± .73	27.16 ± 1.31	38.80 ± 1.65	NCV

Supplementary Table S2. *PHC19* promoter-driven luciferase activity of *cw15* progeny in response to g-lysin treatment. Luciferase activity is expressed as relative light units (RLUs) based on luminescence quantification. T1-1 and T1-3 did not have the *PHC19*-luciferase construct and were therefore observed only background level luciferase activity. Values are the mean of biological duplicate data.

Tetrad	Luciferase activity (RLU)		Genotype	
	- g-lysin	+ g-lysin	*PHC19-gLUC*	*cw15*
T1-1	88	118	no	mutant
T1-2	1998	1781	yes	mutant
T1-3	61	128	no	wild type
T1-4	197	956	yes	wild type

Supplementary Table S3. Candidate genes for putative cell surface sensors involved in the cell wall integrity signaling of *C. reinhardtii*. *annotation is based on V5.6 available at Phytozome.

Gene_ID*	Name*	Comments	Amino acid length
TRP family group 1: With Pectin_lyase-like repeats (32 ea.)			
Cre01.g001300		HYP-rich	5211
Cre01.g040650			1201
Cre01.g040850			2023
Cre01.g042450		HYP-rich	2673
Cre01.g044350		Leu-binding domain	1559
Cre02.g094801			3739
Cre02.g099055			4106
Cre02.g118700			5013
Cre03.g155700			2773
Cre04.g219150			3718
Cre04.g222650		HYP-rich	3962
Cre04.g227251			2251
Cre06.g265652			4038
Cre06.g305350			1389
Cre06.g309650			3681
Cre06.g309800	MOT2		5696
Cre10.g419200			2898
Cre10.g420600			1297
Cre13.g564151			2404
Cre13.g591300		HYP-rich	5210
Cre14.g619400		HYP-rich	3196
Cre17.g709000		HYP-rich	4275
Cre17.g728450			4007
Containing N-terminal Kelch domain			
Cre01.g052750		HYP-rich	4131
Cre07.g338550		HYP-rich, EF-hand	5889
Cre13.g604050		HYP-rich, EF-hand	3555
Cre17.g706700		HYP-rich	4056
Containing N-terminal SRCR domain			
Cre04.g227500	SRR18		5536
Cre04.g229550	SRR15	HYP-rich	4138
Cre10.g421900	SRR27		3737
Cre12.g552700		HYP-rich	4532

Cre16.g654950	SRR3		4702
TRP family group 2: PKD2-like (13 ea.)			
Cre03.g174100		HYP-rich	4882
Cre11.g475150		partial	362
Cre11.g479383			2673
Cre12.g510350		HYP-rich, PAN domain	6412
Cre12.g515400		HYP-rich, DUF5011 domain	3747
Cre12.g539650		HYP-rich	4829
Cre13.g569550			4409
Cre17.g715300	PKD2		1645
Containing lectin domains			
Cre02.g115400		HYP-rich	4211
Cre06.g309951		HYP-rich	7989
Cre09.g400850	CTL4	HYP-rich, PAN/WSC domain	8145
Cre12.g515350		HYP-rich	5155
Cre16.g682550		HYP-rich, LRR domain	7306
Scavenger receptor cysteine-rich (SRCR) family (22 ea.)			
Cre03.g179450	SRR7	HYP-rich	573
Cre04.g219050	SRR10	HYP-rich, WSC domain	2659
Cre04.g227500	SRR18	TRP domain	5536
Cre04.g229550	SRR15	HYP-rich, TRP domain	4138
Cre08.g383400	SRR11	HYP-rich	2127
Cre08.g383600	SRR21	HYP-rich	2097
Cre09.g404750	SRR2	HYP-rich	990
Cre10.g421900	SRR27	TRP domain	3737
Cre10.g423850	SRR26	HYP-rich	779
Cre12.g552700		HYP-rich, TRP domain	4532
Cre13.g591550	SRR20	HYP-rich	1286
Cre16.g654950	SRR3	TRP domain	4702
Cre17.g706050	SRR17	HYP-rich	4813
Containing N-terminal Trypsin-like domain			
Cre01.g042300	SRR6	HYP-rich	1905
Cre01.g042352		HYP-rich (3000 a.a.)	4951
Cre01.g042502		HYP-rich	3851
Cre01.g045601			1628
Containing C-lectin domains			
Cre01.g002787	SRR24A	HYP-rich, GH18 domain	4347
Cre05.g245352		HYP-rich, PAN domain	5227
Cre10.g431050	SRR13	HYP-rich, PAN domain	1874
Cre10.g458350	SRR16	HYP-rich, GH18 domain	9052

Cre14.g631100	SRR29	HYP-rich, WSC/LysM domain	2403
MscS-like family (6)			
Cre01.g035750	MSC3		706
Cre01.g055432		HYP-rich	1524
Cre07.g329300	MSC1	Chloroplast-localized	493
Cre10.g453400	MSC4	HYP-rich, EF-hand	2899
Cre12.g505800	MSC6	EF-hand	1240
Cre13.g591100	MSC5	HYP-rich, EF-hand	2362
OSCA1 (DUF221) family (11)			
Cre08.g360400	ERM1		1370
Cre08.g360500	ERM2		1235
Cre08.g360564			1217
Cre08.g360600	ERM4		1482
Cre09.g399912			1549
Cre10.g460150			1558
Cre12.g532151	ERM7		2129
Cre12.g560050	ERM12	HYP-rich	3083
Cre13.g574850	ERM6		2110
Cre13.g579400	ERM8		2239
Cre17.g738150	ERM10		1887

Supplementary Table S4. Primers used in this study

Gene	Forward primer	Reverse primer
RHM1	ACGGGCTCAAGAAGACCATC	GTTTAGTTGGCCAGGAATGG
AraGT1	AGGTGCTGCACTTCACATCC	GTCTTGGTATCCCAGGTTCC
SEC61G	CACCAGCAACCATGGATC	CTTAGGAGCTCATGATGAC
PHC19	GCAGATATAGGGTGGGACGC	CCCTGGATCGTTCCCTTCTG
GAS28	AATCCATGCCGTACCAAGCA	CCTATGTGCGGACCTAGCAG
GAS30	ATGCCCGCAACAGTTACAGA	GCCCGAGGTTCTTGCTACTT
GAS31	CTGGCCACCTTCCCTTACTG	ACGCCGGTGTTGTATTGAGT
RACK1	GCCACACCGAGTGGGTGTCGTGCG	CCTTGCCGCCCGAGGCGCACAGCG

Supplementary Figure S1. Hypothetical signaling mechanisms for the g-lysin-induced responses. Illustrations represent a magnified region of the cell near the periphery. Top panel shows the possible elements responding to g-lysin treatment in an inactive (before treatment) condition including: receptor proteins as yellow and red shapes; water flow via contractile vacuole as blue arrows; hydrostatic pressure generated in periplasmic space as green double-headed arrow. Cellular elements are arranged spatially as denoted by CV, contractile vacuole; PM, plasma membrane; CW, cell wall. Bottom panels show active conditions by which cellular elements are stimulated and drive the g-lysin-induced response: (**a**) increased water flow into and out of the cell (increased flux rate); (**b**) receptor-mediated detection of fragmented/digested wall components; (**c**) receptor-mediated detection of physical wall removal.

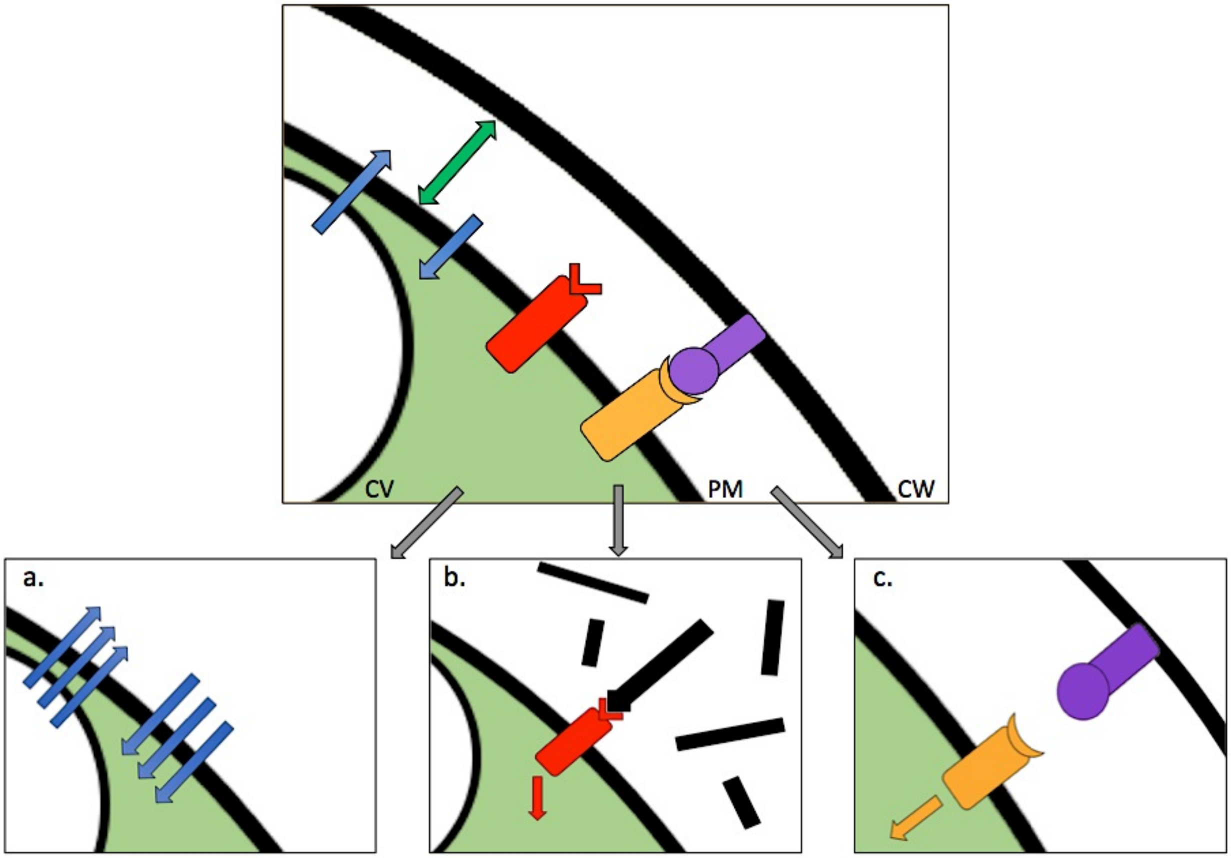

Supplementary Figure S2. Transcript expression suggests modest cellular response to changing osmotic environment. Bar graphs represent the change in transcript activity in half-diluted liquid media (1/2 TAP, black bars) and liquid media plus sucrose (TAP +SS, medium grey bars) compared to the normal TAP media (light grey bars). mRNA levels are quantified as fold change of the TAP condition. Error bars represent one standard deviation from the mean of biological duplicate samples. Welch's t-test indicates statistical significance at $p \leq 0.05$ (*), $p \leq 0.001$ (***), $p \leq 0.0001$ (****); $\alpha = 0.05$.

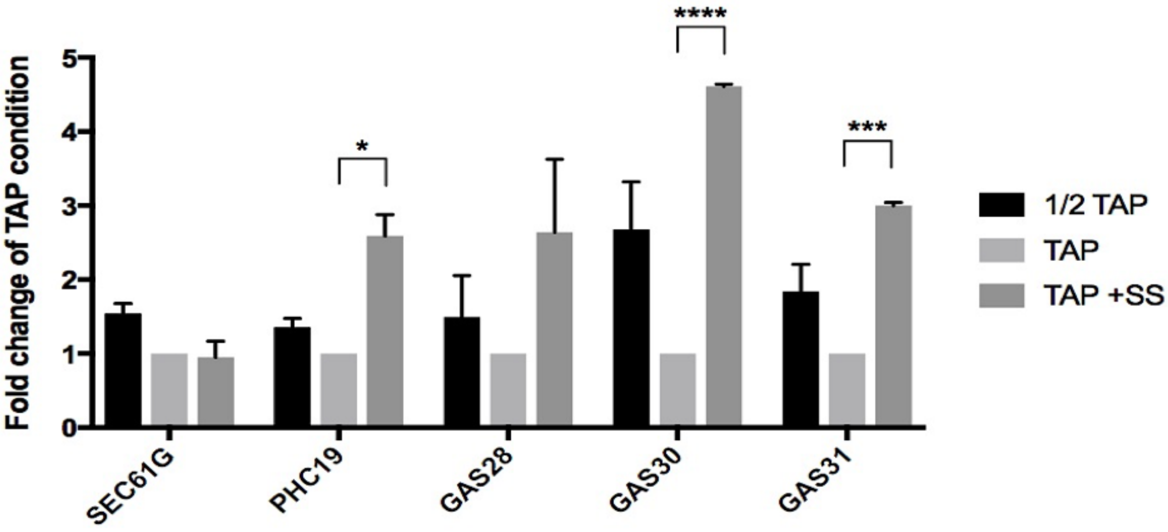

Supplementary Figure S3. *cw15* progeny showed constitutive CW gene expression. (**a**-**d**) Bar graphs represent the change in gene expression for *PHC19* (a), *GAS28* (b), *GAS30* (c), and *SEC61G* (d) in *cw15* cells. Untreated control samples are represented by black bars, grey bars represent cells treated with g-lysin. Gene expression is quantified in terms of fold change compared to the untreated *cw15* samples. Error bars represent one standard deviation from the mean of biological triplicate samples. Welch's t-test indicates statistical significance at $p \leq 0.05$ (*), $p \leq 0.01$ (**); $\alpha = 0.05$.

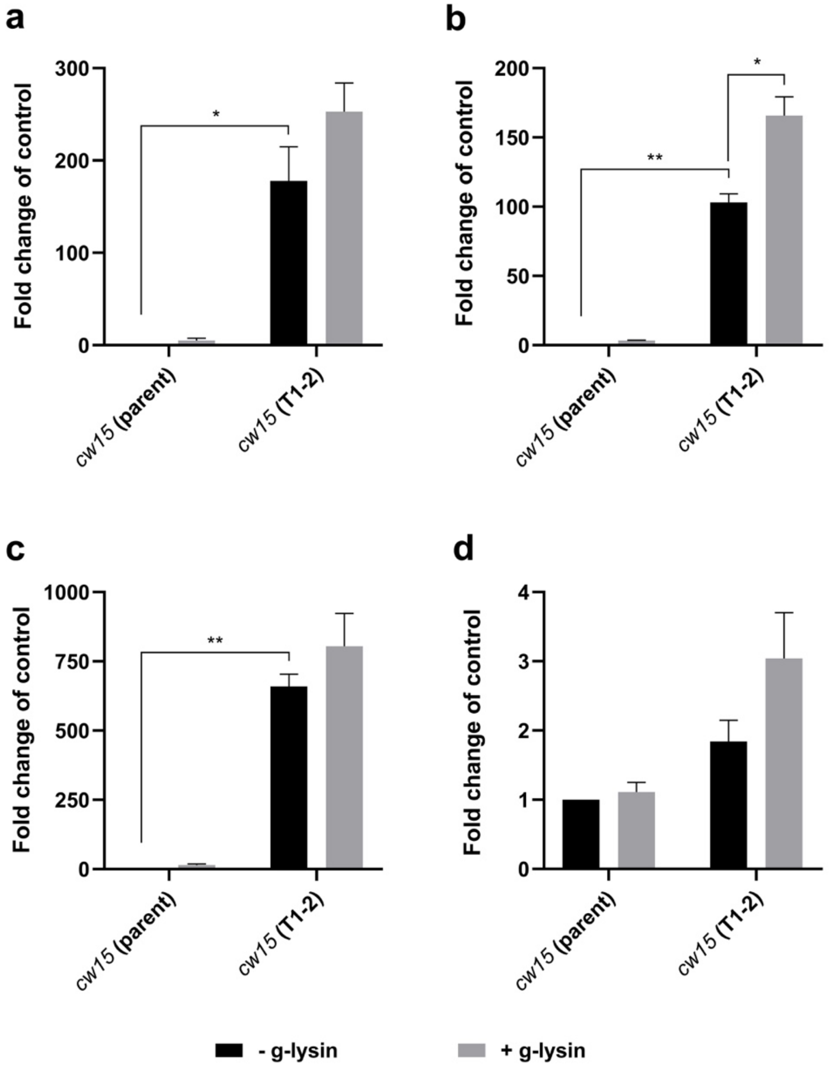

Supplementary Figure S4. Diagrammatic representation of osmoregulation in *C. reinhardtii* cells. (**a)** 2D visualization of water flow through cells near the periphery. Important cellular structures labelled: CV, contractile vacuole; PS, periplasmic space; PM, plasma membrane; CW, cell wall; ECS, extracellular space. Water flux in/out of cell represented by blue arrows. Pressure potentials represented by orange arrows; ψ_{Tp}, turgor pressure; ψ_{Hp}, hydrostatic pressure. Solute transporter represented by pink shape and arrow. (**b**) Equation and derivation for total cellular water flux: A, surface area (m^2); P_f, permeability coefficient (m/s); ψ_w, water potential (Pa); ψ_s, solute potential (Pa); ψ_p, pressure potential (Pa); ψ_g, gravitational potential (Pa); ψ_m, matrix potential (Pa).

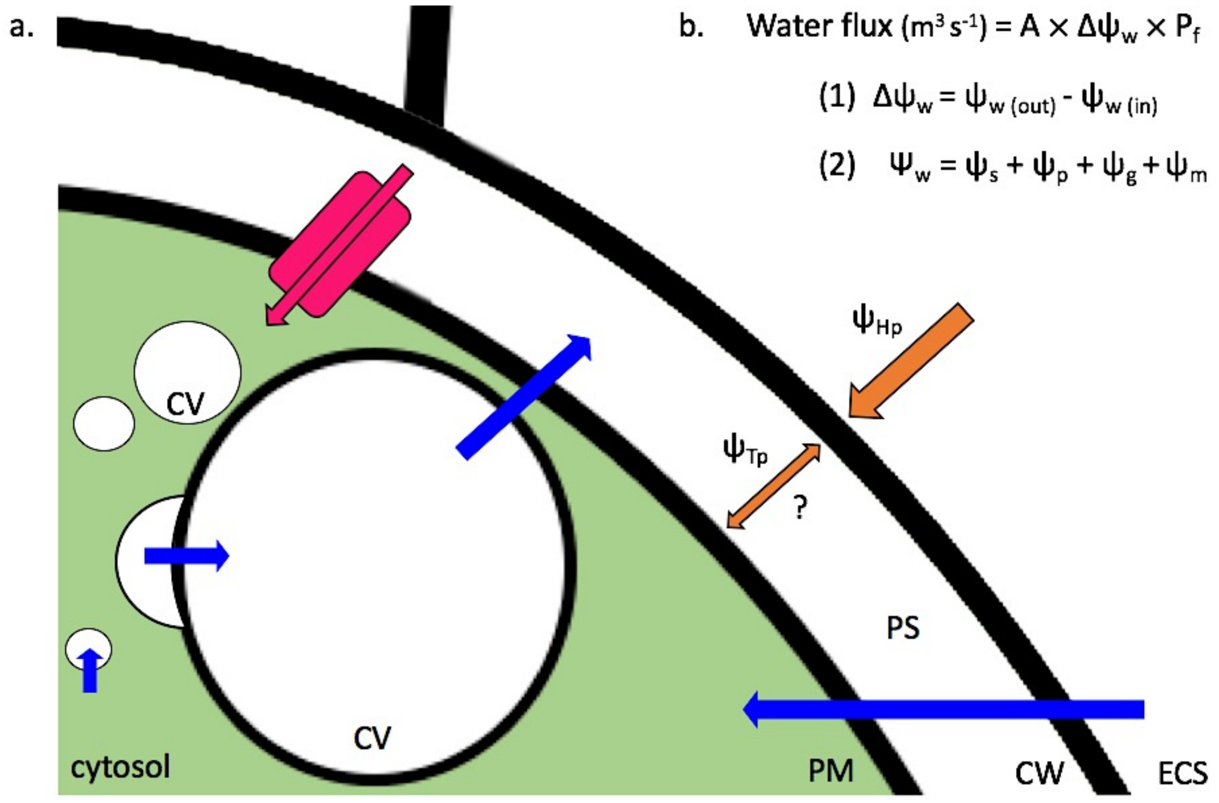

Supplement: Supplementary file 1 — Supplementary Text, Tables and Figures [file 41598_2019_48523_MOESM1_ESM.pdf]
